# On the Order of the Fractional Laplacian in Determining the Spatio-Temporal Evolution of a Space-Fractional Model of Cardiac Electrophysiology

**DOI:** 10.1371/journal.pone.0143938

**Published:** 2015-12-02

**Authors:** Nicole Cusimano, Alfonso Bueno-Orovio, Ian Turner, Kevin Burrage

**Affiliations:** 1 Australian Research Council Centre of Excellence for Mathematical and Statistical Frontiers, Queensland University of Technology, Brisbane, Queensland, Australia; 2 Department of Computer Science, University of Oxford, Oxford, United Kingdom; University of Minnesota, UNITED STATES

## Abstract

Space-fractional operators have been used with success in a variety of practical applications to describe transport processes in media characterised by spatial connectivity properties and high structural heterogeneity altering the classical laws of diffusion. This study provides a systematic investigation of the spatio-temporal effects of a space-fractional model in cardiac electrophysiology. We consider a simplified model of electrical pulse propagation through cardiac tissue, namely the monodomain formulation of the Beeler-Reuter cell model on insulated tissue fibres, and obtain a space-fractional modification of the model by using the spectral definition of the one-dimensional continuous fractional Laplacian. The spectral decomposition of the fractional operator allows us to develop an efficient numerical method for the space-fractional problem. Particular attention is paid to the role played by the fractional operator in determining the solution behaviour and to the identification of crucial differences between the non-fractional and the fractional cases. We find a positive linear dependence of the depolarization peak height and a power law decay of notch and dome peak amplitudes for decreasing orders of the fractional operator. Furthermore, we establish a quadratic relationship in conduction velocity, and quantify the increasingly wider action potential foot and more pronounced dispersion of action potential duration, as the fractional order is decreased. A discussion of the physiological interpretation of the presented findings is made.

## Introduction

Excitable media models are typical mathematical tools used to reproduce *in silico* the generation and spread of electrical signals across biological excitable tissue, such as cardiac or neural tissue. These mathematical models are generally defined as systems of differential equations combining information at the microscopic level (on the response of a single excitable cell to an applied electrical stimulus), with information on the dynamics of the signal propagation at the tissue level.

Classical equations describing electrical propagation in space at a macroscopic level are based on modelling strategies that represent the tissue as a continuous medium characterised by space average quantities according to the homogenisation principle [1], that is, under the assumption that the complexity of the composite structure observed at the microscale has a negligible effect on the propagation of electrical signals at the macroscale. In the case of cardiac tissue, as discussed by Clayton et al. [2], the use of the homogenisation principle has well-established limitations in representing the real nature of the tissue and the effects of its highly heterogeneous microstructure on modulation of signal conduction.

The spatial complexity and heterogeneity of the structure in which an observed transport phenomenon takes place might lead to significant deviations from the standard laws of diffusion [3]. In these settings, classical differential equations fail to reproduce the features of the observed anomalous transport behaviour and fractional models involving non-integer order differential operators have been proposed as an alternative modelling approach in many practical appliactions ranging from the study of rotating flows [4], hydrology [5], fluid dynamics [6] and molecular diffusion in porous media [7], to medicine [8], biology [9], ecology [10], and many others.

To the best of our knowledge, the work by Bueno-Orovio et al. [11] is the first example of the use of a space-fractional mathematical model in cardiac electrophysiology. In [11], the authors implement a fractional modification of a mathematical model of electrical signal propagation through cardiac tissue and successfully show that the fractional model is able to capture important features of experimentally recorded data better than the corresponding standard (non-fractional) formulation. The biophysical justification behind the use of such a fractional operator for this particular application is based on potential electric field theory. As discussed in [11], the inhomogeneities present on a variety of length scales in biological tissue give rise to secondary sources that add up to the primary source field corresponding to the assumption of a uniform and infinite volume conductor. These secondary sources can be seen as a dipole modulation of the electrical potential associated with a point source in a homogeneous tissue (monopole), and by using Riesz potential theory, the authors in [11] showed that a fractional model can be interpreted as a smooth transition between monopole and dipole behaviour, with increasing degree of heterogeneity as the order of the fractional operator *α* → 1 (see [11] for more details).

Bueno-Orovio et al. [11] consider two non-integer values for the order *α* of the space-fractional operator and present their considerations by looking at some physiological quantities of interest, rather than studying the entire solution provided by the fractional model. By observing the results presented in [11], one can sense the presence of particular trends in the solution behaviour produced by varying the parameter *α*. However, without any deeper analysis these ideas cannot be validated nor quantified. If space-fractional excitable media models are to be used in practical situations to account for the heterogeneity present on a high number of scales in the considered excitable tissue, we believe that a better understanding on the role played by the fractional operator and its fractional order has to be achieved.

For these reasons, in this manuscript we propose a systematic study of how the numerical solution of a standard excitable media model is affected by the introduction in the mathematical formulation of the model of a space-fractional operator of non-integer order *α*. Specifically, we aim to investigate (and possibly quantify) the effect produced by using different values of the fractional order *α* on some distinctive features of the solution profile, highlighting crucial differences between the spatio-temporal evolution of the solution in the standard case and the one obtained in the purely fractional case. This work does not involve a direct comparison with experimental data but rather focuses on the numerical aspects of the implementation and computation of the results of standard and space-fractional mathematical models. Nevertheless, we complement our numerical analysis with the discussion of some physiological aspects and implications of the proposed results, providing links to important experimental studies available in the literature.

We begin by introducing the two main components of this work, that is, the monodomain formulation of the Beeler–Reuter cell model and the fractional differential operator of interest, namely the fractional Laplacian (−Δ)^*α*/2^ on an insulated finite domain. After formulating the space-fractional modification of the considered excitable media model, we then describe the methodology adopted in order to compute the numerical solution of the modified system of equations. We present the results of a set of numerical simulations designed in order to investigate how the presence of the fractional operator in space and its order *α* affect the solution of the standard excitable media model. The presented findings are then discussed and our conclusions are drawn.

## Methods

### A particular model of cardiac electrophysiology

Mathematical models of electrical signal propagation in cardiac electrophysiology consist of suitable spatially dependent formulations of specified cell models reproducing the response of a single excitable cell to an applied electrical stimulus. All cell models describe the temporal evolution of the transmembrane potential *v* of the particular excitable cell considered and the changes in *v* caused by the opening and closing of the various ion channels present in the cell membrane, determining the movement of ions into and out of the cell. When an electrical stimulus is applied to a cell, *v* quickly increases (depolarizes) and moves away from its equilibrium value, known as the resting potential. If the applied stimulus is strong enough to push *v* above a certain threshold level, once the stimulus is removed the potential does not immediately return to rest but rather undergoes a large excursion—an action potential (AP)—before eventually reaching the resting state.

In order to account for pulse propagation, spatial dependence is typically introduced via the bidomain formulation of the considered cell model (for example, see [12]). The physiologically grounded bidomain model is often replaced in practice by a less computationally demanding formulation [13], known as the monodomain, based on some simplifying assumptions on the conductivity properties of the tissue in which electrical propagation occurs. In this work we focus on the monodomain formulation of the cell model originally developed by Beeler and Reuter [14] in the study of the excitability properties of ventricular myocardial fibres. The mathematical model considered can be written as the following coupled system:

$$\chi \left(C_{m}\frac{\partial v}{\partial t}+I_{\text{ion}}\left(v,z\right)\right)-\nabla \cdot \left(D\nabla v\right)=I_{\text{stim}}$$ (1)

$$\frac{dz}{dt}=f(v,z),$$ (2)

where *C*_*m*_ is the membrane capacitance per unit area, *χ* is the cell surface-to-volume ratio, **z** is a suitable vector of secondary variables used in the description of the dynamics of the ion channels in the cell membrane, *I*_ion_ is the sum of all transmembrane ionic currents, *D* is an effective conductivity tensor, and *I*_stim_ is the electrical stimulus.

The specification of the vector **z**, the vector-valued function **f** right-hand side of Eq (2), and the analytic expression of *I*_ion_ in Eq (1) depend on the particular type of cell model considered and the level of detail that one wants to incorporate into the cell model. The Beeler–Reuter (BR) model is probably one of the simplest cell models that captures the correct form of the cardiac AP of ventricular cells in a satisfactory and meaningful way, and its mathematical formulation consists of a system of eight ordinary differential equations (see S1 Appendix for the complete model description). Considering a higher level of detail in the cell model and the corresponding bidomain formulation (as in the case of state-of-the-art models of electrical propagation in cardiac tissue) comes at a considerable computational cost and is outside of the scope of the presented study. However, the numerical methodology proposed here can be easily generalised to more sophisticated electrophysiological models.

### Fractional Laplacian on an insulated finite domain

Space-fractional differential equations are differential equations in which the classical integer-order differential operators in space are substituted by non-integer counterparts, often referred to as fractional operators. As previously mentioned, differential equations involving non-integer order derivatives have shown to be very powerful tools in the description of transport phenomena whose characteristics substantially deviate from the classical Gaussian and Markovian assumptions and have been adopted as mathematical models for a variety of practical applications.

Among fractional operators in space, the fractional Laplacian (−Δ)^*α*/2^ [15] of order *α* plays an important role due to its probabilistic interpretation. This operator is naturally defined on the entire $R^{n}$, *n* = 1, 2, 3, and only when *α* = 2 one recovers the local character of the definition. However, especially when dealing with applications of practical interest, relationships between the observed quantities often hold only within specified bounded regions $\Omega \subset R^{n}$, and hence suitable mathematical models for these applications must be defined on finite domains.

The restriction of fractional operators to finite domains is by no means trivial and a fundamental role in the definition and implementation of the solution of the problem is played by the particular boundary conditions to which the model is coupled. With fractional operators the mere specification of a local condition at the boundaries is no longer sufficient for the problem to be well-posed, and even though well-established methodologies have been developed for the case of homogeneous Dirichlet boundary conditions (see for example [16]), little is known about all other boundary constraints.

For the particular application considered in this paper, typical boundary conditions are defined under the assumption that the finite domain considered is insulated and hence, in the standard case, they are specified as homogeneous Neumann boundary conditions.

On an insulated one-dimensional domain [0, *L*], the continuous fractional Laplacian (−Δ)^*α*/2^ can be defined via the spectral mapping theorem and the eigenvalue decomposition of the continuous standard Laplacian (−Δ), as originally proposed by Ilić et al. [17, 18]. This definition of the fractional operator correctly represents the insulation properties of the finite domain in both the standard and the fractional cases (as proved by Cusimano [19]) and will be used in this work to formulate the space-fractional modification of the BR monodomain model and to design an efficient solution strategy for the fractional problem considered.

Since the theoretical results obtained in Cusimano [19] have been proved in the one-dimensional case and for *α* ∈ (1, 2], in this study we focus only on this particular range of the fractional order and on one spatial dimension, leaving the generalisation of the proposed study to higher dimensions as future extension of this work.

### Space-fractional Beeler–Reuter model

Let us consider the BR monodomain formulation on an insulated finite interval [0, *L*], that is, system Eqs (1)–(2) in which *I*_ion_, **z** and **f** are given by the BR cell model as defined in S1 Appendix. We introduce the following space-fractional modification of system Eqs (1)–(2):

$${\chi \left(C_{m}\frac{\partial v}{\partial t}+I_{\text{ion}}\left(v,z\right)\right)+D\left.(-\Delta \right)}^{\alpha /2}v=I_{\text{stim}}$$ (3)

$$\frac{dz}{dt}=f(v,z),$$ (4)

where *D* is now simply a constant and (−Δ)^*α*/2^ is the one-dimensional fractional Laplacian of order *α* ∈ (1, 2] on [0, *L*], defined via its spectral expansion and embedding in its definition the correct representation of insulating boundary conditions at both ends of [0, *L*]. A physiological justification for the use of fractional diffusion models in the description of structurally heterogeneous excitable media, as a representation of the modulation of the electrical field of a homogeneous conductor by the secondary electrical sources associated with tissue inhomogeneities, has been presented in [11].

Specifically, (−Δ)^*α*/2^ is the continuous operator with eigenfunctions ${\left\{\varphi_{j}\right\}}_{0}^{\infty }$, defined for *x* ∈ [0, *L*] as

$$\varphi_{j}\left(x\right)=w_{j}\cos\left(\frac{j\pi x}{L}\right),$$ (5)

with $w_{0}=1/\sqrt{L}$, $w_{j}=\sqrt{2/L}$ for *j* ≥ 1, and corresponding eigenvalues ${\left\{\lambda_{j}^{\alpha /2}\right\}}_{0}^{\infty }$, that is, the fractional powers of the eigenvalues of the standard continuous Laplacian (−Δ), coupled to homogeneous Neumann boundary conditions on [0, *L*], defined as $\lambda_{j}={\left(\frac{j\pi }{L}\right)}^{2}$ for all *j* ≥ 0.

Throughout the rest of the paper we assume that the cell model description in system Eqs (3)–(4) is given by the BR model. However, most of the considerations made in this section do not depend on the particular specification of *I*_ion_, **z**, **f**, and hence could be easily extended to the monodomain formulation of any other cell model of interest.

### Solution methodology

In order to compute the numerical solution of system Eqs (3)–(4) on a given temporal interval [0, *t*_*f*_], with *t*_*f*_ > 0, we adapt the strategy proposed by Whiteley [20] for the standard monodomain formulation of an excitable media model and combine it with a spectral approach for the solution of the space-fractional reaction-diffusion Eq (3). The main idea behind Whiteley’s approach [20] is to introduce a temporal grid for [0, *t*_*f*_] and starting from a given initial condition, to compute the numerical solution at each time iteration in two steps. First, the value of the transmembrane potential *v* is updated by solving Eq (3), while keeping the value of **z** fixed at the previous time iteration. Second, the vector **z** is updated by solving the system of differential Eq (4) in which the vector-valued function **f** has been evaluated by using the newly computed value of *v*.

The only difference between the strategy presented here and the one proposed by Whiteley [20] for the standard case is the way in which the value of the transmembrane potential *v* is updated at each time step. Here, to compute the solution of Eq (3) we exploit the spectral definition of the fractional Laplacian and extend the spectral approach presented in [19] to the case of a space-fractional reaction-diffusion equation (similarly to that done by Bueno-Orovio et al. [21]).

In particular, we look for the solution *v*(*x*, *t*) as a linear combination of the orthonormal eigenfunctions of the one-dimensional fractional Laplacian (−Δ)^*α*/2^ on the insulated domain, that is, in the form $v\left(x,t\right)=\sum_{j=0}^{\infty }\;{\hat{v}}_{j}\left(t\right)\varphi_{j}\left(x\right)$, where each ${\hat{v}}_{j}\left(t\right)$ is the integral coefficient ${\hat{v}}_{j}\left(t\right)=\int_{0}^{L}\;v\left(x,t\right)\varphi_{j}\left(x\right)\;dx$ (these eigenfunctions can be shown to form a basis for a suitable Hilbert space in which the solution to the problem is sought—see for example Hutson et al. [22] for more details). Let us introduce a grid of time points *t*_*k*_ with uniform time step Δ*t*. Let *v* ^*k*^: = *v*(*x*, *t*_*k*_), and **z** ^*k*^: = **z**(*x*, *t*_*k*_). The solution of Eq (3) is computed at each time step via the semi-implicit Euler scheme in which the spatial derivative of *v* is evaluated at *t*_*k*+1_, while the ionic term is considered fixed at *t*_*k*_, that is, by solving

$$C_{m}\frac{v^{k+1}-v^{k}}{\Delta t}=-\frac{D}{\chi }{\left(-\Delta \right)}^{\alpha /2}v^{k+1}-I_{\text{ion}}\left(v^{k},z^{k}\right)+\frac{1}{\chi }I_{\text{stim}}.$$ (6)

By using the spectral definition of the fractional Laplacian, that is, the fact that ${\left(-\Delta \right)}^{\alpha /2}v=\sum_{j=0}^{\infty }\lambda_{j}^{\alpha /2}{\hat{v}}_{j}\varphi_{j}$, and due to the orthonormality of the eigenfunctions *φ*_*j*_, the computation of *v* ^*k*+1^ can be fully diagonalised and each integral coefficient can be updated independently at each time step as follows:

$${\hat{v}}_{j}^{k+1}=\frac{1}{1+\Delta t\frac{D}{\chi \;C_{m}}\;\lambda_{j}^{\alpha /2}}\left[{\hat{v}}_{j}^{k}+\Delta t\;{\hat{g}}_{j}\left(v^{k},z^{k}\right)\right],$$ (7)

where once again $\left\{\lambda_{j}^{\alpha /2}\right\}$ is the set of eigenvalues of the fractional operator (−Δ)^*α*/2^ and ${\hat{g}}_{j}$ is the *j*-th integral coefficient of the spectral expansion of the source term $g\left(v,z\right):=\frac{1}{C_{m}}(-I_{\text{ion}}\left(v,z\right)+\frac{1}{\chi }I_{\text{stim}})$ evaluated at *v* ^*k*^ and **z** ^*k*^.

Once the updated value of *v* has been computed, for the vector of secondary variables **z** we adopt Whiteley’s method without the need of any modification and hence consider a fully implicit Euler scheme to solve the ODE system Eq (4). Due to the particular form of the right-hand side of the differential equation of six out of seven of the secondary variables in the BR model (linear in the considered component of the vector **z** and independent from all other secondary variables—see S1 Appendix), the backward differentiation formula allows us to obtain an unconditionally stable explicit expression for the updated value. When the right-hand side of the equation is not linear, as in the case of the calcium concentration for the BR cell model, an inexact Newton method (in which the Jacobian is “frozen” at the first iteration) is used instead.

Note that for practical reasons the eigenfunction expansion of the solution and the source term must be truncated after a finite number of terms, and the integral coefficients ${\hat{v}}_{j}^{k}$ and ${\hat{g}}_{j}^{k}$ must be somehow approximated. Let us consider a uniform spatial grid of *N* + 1 nodes *x*_*i*_ = *ih*, for the discretisation of the insulated finite domain [0, *L*], with $h=\frac{L}{N}$, and *i* = 0, 1, …, *N*. In all our numerical simulations we will consider the first *N* + 1 eigenfunctions of the spectral expansion and approximate all integral coefficients by using the trapezoidal rule on the considered nodes. This approach can be shown to be equivalent to computing the solution on [0, *L*] via the discrete cosine transform, assuming symmetry with respect to both ends of the spatial interval and periodicity with period 2*L* for the extended solution defined on R.

## Results

In this section we provide a set of numerical results aimed at studying the effect of reducing the order *α* on the behaviour of the solution produced by the space-fractional modification of the BR monodomain model.

Let us consider the spatial domain [0, *L*] with *L* = 1 cm, the temporal interval [0, *t*_*f*_] with *t*_*f*_ = 500 ms, and a uniform time step equal to Δ*t* = 0.01 ms. Unless otherwise stated, throughout this section we assume that *C*_*m*_ = 1*μ*F⋅cm^−2^, *χ* = 1400 cm ^−1^, and *D* = 1 mS⋅cm^−1^. The transmembrane potential is assumed to be initially equal to *v* = −85mV on the entire spatial domain. Similarly, the dimensionless value of the first six variables in **z** is given initially by the vector [0, 1, 1, 0, 1, 0] at each spatial node, and the scaled calcium concentration (that is, the seventh secondary variable of the model) is assumed to be initially equal to 1 mol⋅l^−1^ everywhere.

Each simulation proposed here was run for a certain temporal interval [0, *t*_stim_] without the application of any external stimulus so that the steady state of each variable could be reached before any AP was triggered. The electrical stimulus was applied at *t*_stim_ to a small region $\left[0,\bar{x}\right]$ surrounding the origin of the domain for two consecutive milliseconds. Its strength was set equal to twice the diastolic threshold computed in the standard case (*α* = 2) and defined as the minimum current that produces a successful propagation of the electrical impulse. In particular, by applying an electrical stimulus *I*_stim_ = 40 *μ*A⋅cm^−3^ on the sub-interval $\left[0,\bar{x}\right]$, $\bar{x}=0.05\;\text{cm}$, at *t*_stim_ = 10 ms for two consecutive milliseconds, we are able to trigger the propagation across the domain of an AP.

### Convergence

In order to assess convergence of the numerical solution produced by our solution method and to identify acceptable values of the key parameters *N* and Δ*t* for our numerical simulations, we follow a similar approach to the one proposed by Pathmanathan et al. [12]. We consider successive refinements of a key parameter *q* (either *q* = *N* or *q* = Δ*t*) and compare the resulting solutions via four relative metrics against a reference solution (computed with the finest spatial mesh, smallest uniform time step). Specifically, we consider the relative Euclidean norm of the temporal profile of the solution computed at a selected node *P*_*i*_ ∈ [0, *L*], the relative error in the AP duration at 50% and 90% repolarization at the node *P*_*i*_ (APD_50_ and APD_90_, respectively), and the relative change in the conduction velocity (CV) measured as the ratio of the distance between two nodes of [0, *L*] and the difference in the corresponding activation times. Activation time of a given node was defined as the time when the voltage reached 10% of its full depolarization. APD_50_ and APD_90_ were defined as the time intervals between activation and the time when the voltage (at the considered point) reached 50% and 90% of repolarization to its resting value, respectively. In both cases, linear interpolation was used to obtain better resolved time values.

**Fig 1:**
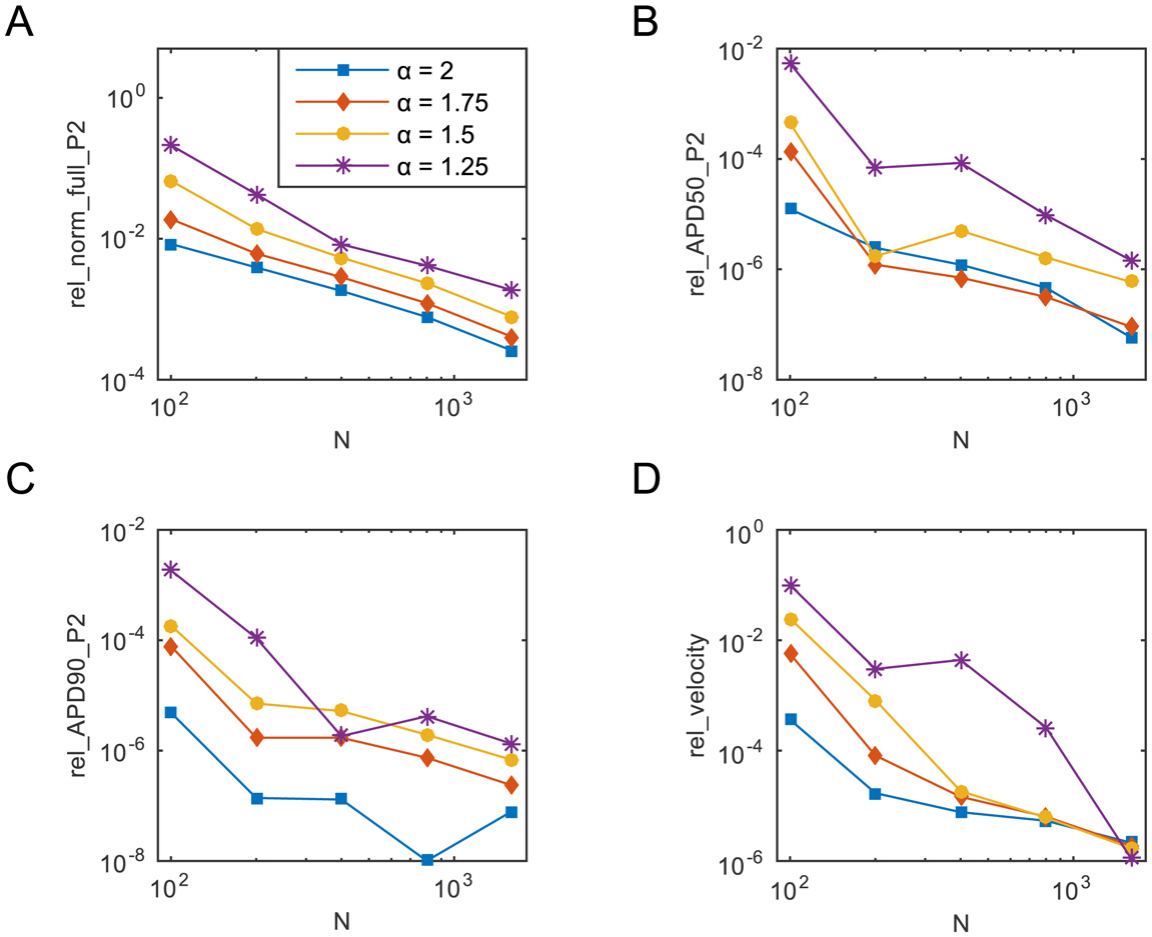
The four relative metrics as a function of the key parameter *N* (the number of eigenfunctions), for four selected values of *α*. The solution is computed for all values of *α* on the time interval [0,500 ms] with a uniform time step Δ*t* = 0.01 ms. All relative errors are evaluated by comparing the solution for a specific combination of *α* and *N* with the solution obtained for the same *α* when *N* = 3200. In all simulations, the stimulus *I*_stim_ = 40 *μ*A⋅cm^−3^ was applied on [0,0.05 cm] at *t*_stim_ = 10 ms for two consecutive milliseconds and then removed.

In order to compute the quantities of interest for our metrics, we focus on three equally spaced nodes, namely *P*_1_(*x* = 0.25), *P*_2_(*x* = 0.5), and *P*_3_(*x* = 0.75). In Fig 1, we display the above mentioned metrics as a function of the key parameter *N* for four different values of *α*. For each *α* we use as reference solution the solution computed with the considered *α* on a grid of 3201 spatial nodes.

Fig 1(A), 1(B) and 1(C) display the value of the relative Euclidean norm and the relative APD_50_ and APD_90_ only at the mid-point *P*_2_ but very similar qualitative results were obtained for these metrics also at *P*_1_ and *P*_3_ (results not shown). In order to compute the relative velocity of Fig 1(D) we use the activation times of and the distance between *P*_1_ and *P*_3_.

As is clearly visible in Fig 1(A), as *N* increases, for all values of *α* the approximation of the solution over the entire temporal interval improves. However, as we reduce *α*, a larger value of *N* is necessary in order to achieve a given accuracy. The other three metrics exhibit a similar overall trend, even though the error decay is not always monotonic and in some instances for a fixed *N* a lower value of the relative error is obtained when using a smaller *α*.

In all simulations performed to compute the errors displayed in Fig 1 we considered Δ*t* = 0.01 ms and only varied *N*. There is a certain amount of *a posteriori* analysis in this choice and the particular value chosen is the result of a series of simultaneous refinements of both key parameters, *N* and Δ*t*.

**Fig 2:**
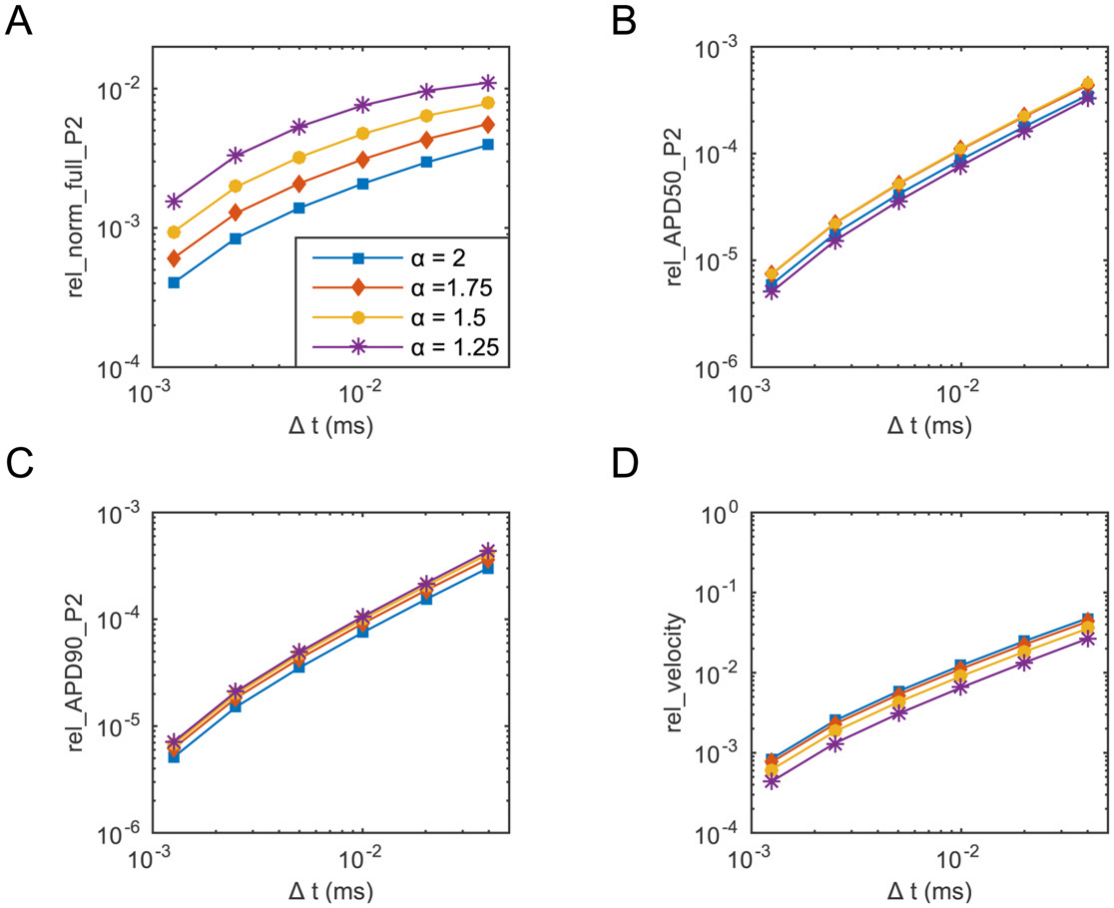
The four relative metrics as a function of the key parameter Δ*t* (the time step), for four selected values of *α*. The solution is computed for all values of *α* on the time interval [0,500 ms] with *N* = 400. All relative errors are evaluated by comparing the solution for a specific combination of *α* and Δ*t* with the solution obtained for the same *α* when Δ*t* = 6.25⋅10^−4^ ms. In all simulations, the stimulus *I*_stim_ = 40 *μ*A⋅cm^−3^ was applied on [0,0.05 cm] at *t*_stim_ = 10 ms for two consecutive milliseconds and then removed.

Fig 2 shows the behaviour of the four metrics as a function of the key parameter Δ*t*, for the same four values of *α*. Clearly, as Δ*t* decreases the error in all relative metrics becomes smaller. However, similarly to that observed in Fig 1, for a fixed value of the key parameter Δ*t*, the corresponding value of rel_norm_full_P_2_ increases as *α* is reduced, indicating the need for refining the temporal mesh as *α* is reduced to achieve a given level of accuracy in the numerical approximation. The effect of *α* on the other three metrics is less significant. As the time step is varied, the overall trend exhibited by these three metrics is indeed quite similar and for a given Δ*t* the magnitude of the error is not affected by the use of different values of *α*. Once again, we computed the value of the first three metrics also at *P*_1_ and *P*_3_ obtaining very similar results (not shown).

In light of the proposed convergence results, we decided to set *N* = 400 (corresponding to a mesh size equal to *h* = 0.0025 cm) and Δ*t* = 0.01 ms. With this choice of key parameters we observe that rel_norm_full ≤10^−2^ for all the considered values of *α* in Fig 1, meaning that the percentage error in the solution approximation is smaller than 1% over the entire temporal interval [0, *t*_*f*_]. Similarly, from Fig 2 we see that the error computed in the calculation of the CV with this choice of key parameters is smaller than 1% as well. Finally, Fig 2(B) and 2(C), show that when *N* = 400 the percentage error in the computation of both the APD_50_ and APD_90_ is smaller than 0.01% and hence, ensures that the value of these two biomarkers is computed with an even higher precision.

### Effects of a reduced *α* on AP shape and propagation across the domain

In this section we investigate the effect of reducing the value of the fractional order *α* on the spatio-temporal propagation of the excitation wave triggered by the applied stimulus.

#### Temporal effects

**Fig 3:**
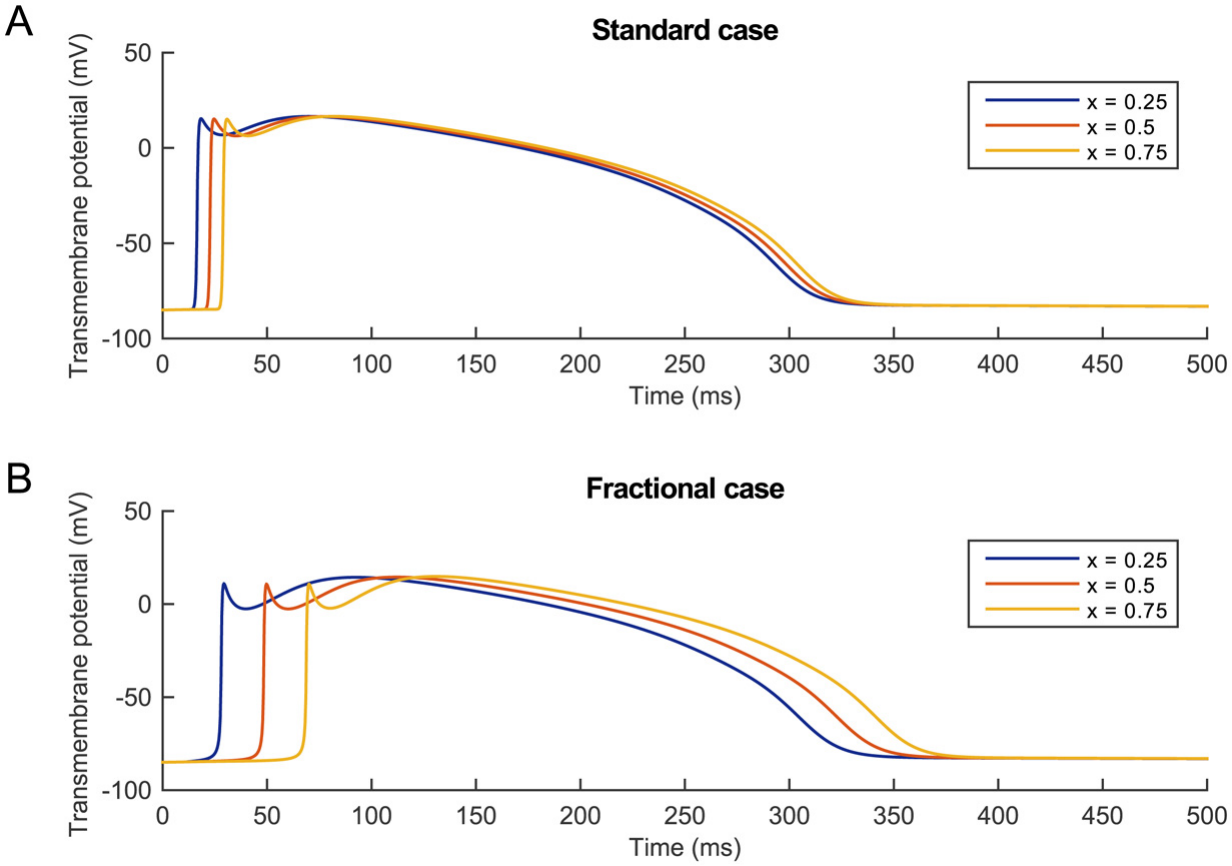
Temporal profile of the solution for *t* ∈ [0,500 ms] at three equally spaced nodes *P*_1_ (*x* = 0.25), *P*_2_ (*x* = 0.5), *P*_3_ (*x* = 0.75) of the spatial domain [0, 1 cm]. (A) Solution obtained when *α* = 2. (B) Solution obtained when *α* = 1.5. In both cases the stimulus *I*_stim_ = 40 *μ*A⋅cm^−3^ was applied on [0,0.05 cm] at *t*_stim_ = 10 ms for two consecutive milliseconds and then removed.

We begin by studying how changes in *α* modify the temporal profile of the solution and focus on the differences observed in the AP recorded at a given set of points in the spatial domain. Fig 3 shows the AP at *P*_1_, *P*_2_, and *P*_3_ generated by the same applied stimulus *I*_stim_ = 40 *μ*A⋅cm^−3^ for *α* = 2 and *α* = 1.5.

In the standard case depicted in Fig 3(A), we see how the excitation wave triggered by the applied stimulus travels across the spatial domain and produces at each node the AP shape typical of the BR cell model, that is, characterised by the presence of a depolarization peak followed by a small concavity known as an early repolarization, and finally a large dome and a slow recovery to the transmembrane potential resting state. When a fractional value for the parameter *α* is considered (here set equal to *α* = 1.5), the electrical stimulus is still able to trigger an excitation wave travelling across the spatial domain and each considered node, once depolarized, goes through what seems to be the exact same AP (differences discussed below). However, it can be observed that there are interesting differences between the two plots, such as the larger times required for each of the three nodes to be depolarized in the fractional case and the more pronounced early repolarization phase when *α* < 2.

**Fig 4:**
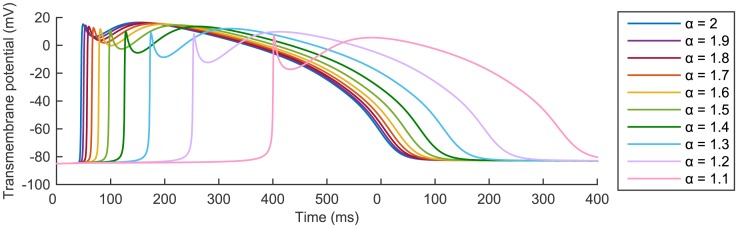
Temporal profile for *t* ∈ [0,500 ms] of the solution obtained with ten different values of *α* at the mid-point of the spatial domain [0, 1 cm]. In all cases the stimulus *I*_stim_ = 40 *μ*A⋅cm^−3^ was applied on [0,0.05 cm] at *t*_stim_ = 10 ms for two consecutive milliseconds and then removed.

In order to better visualise these differences we focus on a single node in space and compare the AP shape generated at this node by *I*_stim_ = 40 *μ*A⋅cm^−3^ for ten different values of *α* ∈ (1, 2]. The results obtained at the mid-point *P*_2_ are plotted in Fig 4. Very similar qualitative results were also found at *P*_1_ and *P*_3_ (results not shown).

As *α* is reduced we observe:

1. a decrease in both the depolarization peak height and the dome peak;
2. a visibly more pronounced early repolarization phase;
3. an increase in the AP foot width (the AP foot is defined as the first portion of the quick AP depolarization);
4. a shift towards the right of the AP, corresponding to a later activation time for the considered node and indicating a reduction in the CV of the propagation pulse.

**Fig 5:**
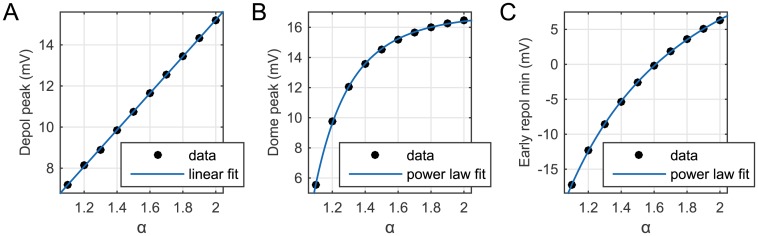
Curve fitting of three data sets obtained from the AP solution profile at the node *P*_2_ for ten different values of *α* ∈ (1, 2]. (A) AP depolarization peak height, (B) dome peak height, (C) early repolarization minimum.

In order to quantify the effects observed qualitatively in Fig 4 and to establish specific relationships between the considered quantities and the fractional order *α*, we then fit the data obtained from our numerical simulations with suitable functions of the parameter *α*. Specifically, in Fig 5(A) we consider the data stored for the AP depolarization peak height and clearly see that a reduction in *α* produces a linear decrease of this quantity. On the other hand, we find that the relationship between *α* and the dome peak (Fig 5(B)) and between *α* and the early repolarization minimum (Fig 5(C)) is nonlinear, and in both cases it can be well described by a power law of the form *f*(*α*) = *aα* ^*b*^ + *c*. The value of the parameters *a*, *b*, *c*, and the goodness of fit statistics for the presented fitting curves is reported in the S2 Appendix.

**Fig 6:**
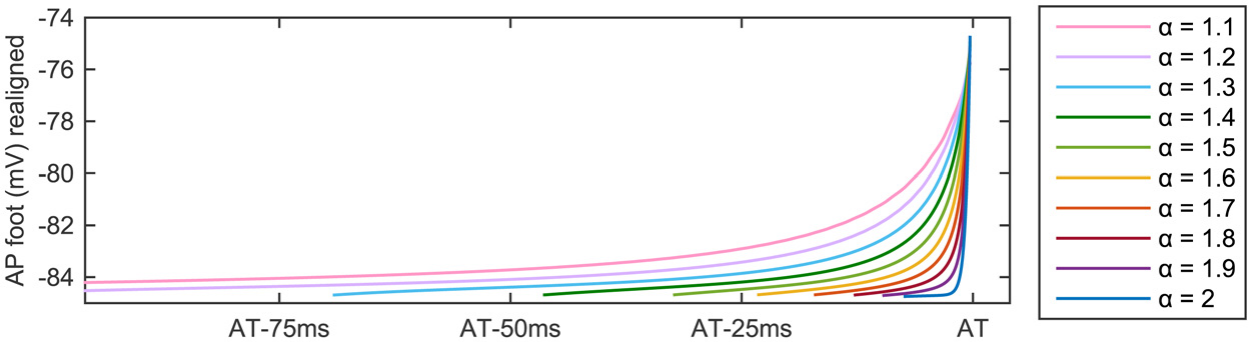
AP foot of the solution profile obtained at the node *P*_2_ for ten different values of *α* ∈ (1, 2]. To aid the visualisation of the differences produced by varying the fractional order, we align the AP foot of the ten solution profiles considered so that the activation time (AT) of the node *P*_2_ coincides for all values of *α*.

A better visualisation of the effects produced by varying the fractional order *α* on the foot of the AP profile at *P*_2_ is given in Fig 6. By aligning the AP obtained with different *α* so that the activation time of each solution profile coincides, we clearly see the change in AP foot width and decrease in curvature as *α* is reduced.

#### Spatial effects

The differences in the activation time of *P*_2_ observed for different values of *α* are a clear indication that the introduction of a fractional order does not only modify the AP profile but also has a sensible effect on the spatial spread of the excitation wave.

**Fig 7:**
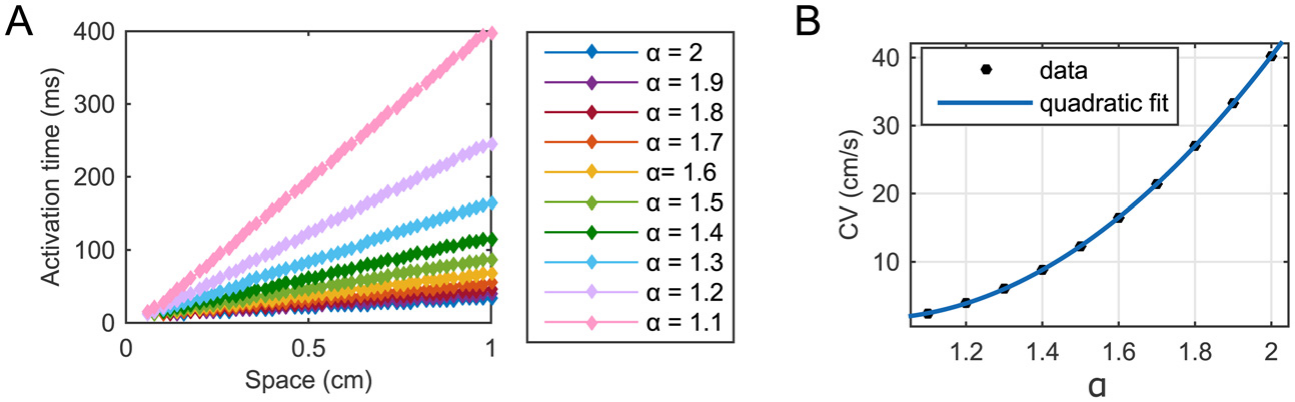
Activation time of forty-eight equally spaced nodes in the spatial domain [0.06 cm, 1 cm] and conduction velocity for ten different values of *α*. The CV corresponding to the reciprocal of the gradient of each of the ten straight lines in (A) was considered as data in (B) and a quadratic dependence of these data points from the fractional order *α* was established.

Fig 3 suggests that for a fixed *α* the depolarization front of the excitation wave triggered by the applied stimulus moves across the spatial interval (away from the stimulus site) with constant speed. In Fig 7(A) we plot the activation time of forty-eight equally spaced nodes in [0, *L*] (we exclude a small part of the domain surrounding the origin to avoid artificial effects due to the applied stimulus), for ten different values of *α* ∈ (1, 2]. We see that the excitation pulse travels linearly in space for all values of *α* (with very slight variations from linearity only in proximity of the boundary *x* = *L*, further away from the stimulus site). The CV of the triggered excitation wave, corresponding to the reciprocal of the gradient of each of these straight lines, is hence constant across the spatial domain. However, its value depends on *α* and decreases as *α* is reduced.

In particular, we find that the relationship between the CV and *α* is not arbitrary, but rather it can be expressed as a quadratic function of *α* as shown in Fig 7(B). Once again, see S2 Appendix for specific values of the parameters involved in the quadratic fit and the corresponding goodness of fit statistics.

#### Dispersion of action potential duration

In this section we make some considerations on the action potential duration (APD), and as a measure of APD we consider the APD_90_ previously introduced in the text. One important characteristic observed in standard simulations of excitable media models is the dependence of the repolarization front advance on the domain geometry and on the proximity to the boundaries [23]. These spatial variations result in differences of APD across the domain already in the standard case. Specifically, an initial reduction in the APD due to the proximity to the stimulus site is followed by the adjustment of the APD about an almost constant level before sensibly reducing in proximity of the insulated boundary *x* = *L*. In order to compare the effect of different values of the parameter *α* on the variations produced in APD across the domain, we measure the APD dispersion (computed here for each *α* as the difference between the value of APD at a given spatial node and the maximum APD value recorded for the same value of *α* over the entire spatial domain).

**Fig 8:**
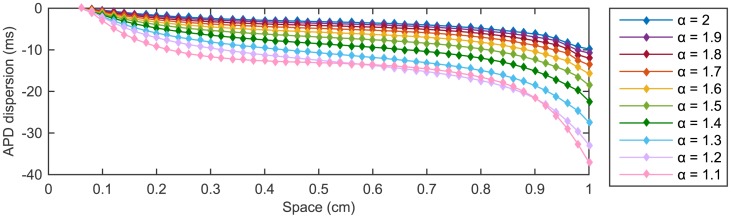
APD dispersion at forty-eight equally spaced nodes in [0.06 cm, 1 cm] for ten different values of *α*. For a fixed *α*, the APD is computed from the AP solution profile recorded at each considered node in the spatial interval. Dispersion of APD is then defined as the difference between the computed value of APD at that node and the maximum APD value recorded for the same value of *α* over the entire spatial domain.

Differences in the APD across the spatial interval are present for all values of *α* (Fig 8). However, as *α* decreases we find a more pronounced effect in the dispersion across an increasing portion of the domain and the reduction in the APD in proximity of the boundary *x* = *L* becomes significantly larger.

The shortening in APD in proximity to the boundary is essentially linked to the finiteness of the spatial domain and to the increase in the repolarization electrotonic current due to the loss of the more depolarized neighbouring cells, as observed by Cherry and Fenton [23]. When *α* < 2, we add to the reflective nature of the insulated boundary the fractional character of the solution. Hence, as the repolarization front moves closer to the end of the spatial interval, the influence on the solution of the increasing number of more repolarized nodes (closer to the stimulus site) becomes stronger as *α* decreases. Therefore, the acceleration produced on the repolarization becomes greater and the shortening of APD more marked.

We stress that the presented results on APD dispersion are not a simple effect due to the small domain size chosen for our simulations. In fact, when longer cables are considered dispersion of APD is still present and becomes increasingly pronounced as the fractional parameter *α* is reduced (results not shown).

#### Reduced *α* versus reduced conductivity

**Fig 9:**
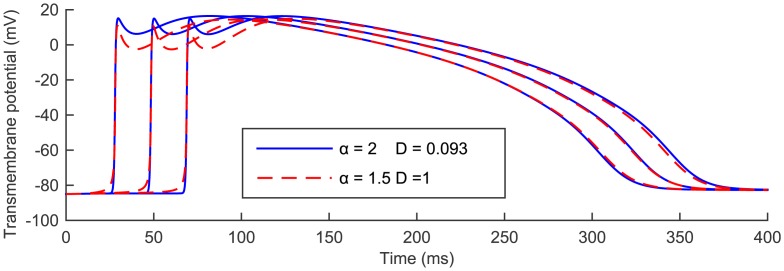
Temporal evolution for *t* ∈ [0,400 ms] of the standard solution with reduced conductivity and the fractional solution with unit conductivity parameter at three equally spaced nodes *P*_1_, *P*_2_, *P*_3_, in the spatial domain [0, 1 cm]. The continuous blue line corresponds to the standard solution obtained with *α* = 2, *D* = 0.093 mS⋅cm^−1^. The dashed red line represents the fractional solution obtained by setting *α* = 1.5, *D* = 1 mS⋅cm^−1^. In both cases the stimulus *I*_stim_ = 40 *μ*A⋅cm^−3^ was applied on [0,0.05 cm] at *t*_stim_ = 10 ms for two consecutive milliseconds and then removed.

The introduction of a fractional operator in space in the BR monodomain formulation resulted in the solution of the model being characterised by a reduction of the CV and various changes in a number of other features of the AP shape. Clearly, a reduction in the CV could be obtained also by considering the standard BR monodomain formulation (*α* = 2) and simply reducing the conductivity parameter *D* in Eq (3). We conclude this section by providing a comparison between the solution obtained from the space-fractional modification of the BR monodomain model for a given fractional index and the one found by reducing the conductivity coefficient in the standard case in order to match the value of the CV obtained in the fractional case. Specifically, in Fig 9 we consider the case *α* = 1.5, *D* = 1 mS⋅cm^−1^, generating an excitation wave with CV = 12.2903 cm⋅s^−1^, and compare the AP generated at the usual three equidistant nodes *P*_1_, *P*_2_, *P*_3_, to the solution obtained at the same nodes when *α* = 2 and *D* = 0.093 mS⋅cm^−1^.

Even though the choice of these two combinations of parameters *α* and *D* was made so that the CV of the corresponding solutions coincided, once again we see that the fractional solution (red) and the standard solution (blue) are quite different in the AP shape. In fact, the fractional solution is characterised by a wider AP foot, a lower depolarization peak, an accentuated early repolarization phase, and a slightly lower dome peak, highlighting the fundamentally different nature of the two cases considered. We also see that at *P*_3_ the distinction between the repolarization phase of the AP generated in the standard and the fractional cases becomes visible, identifying also the more pronounced shortening of APD as another distinctive feature of the fractional modification of the model. Achieving a larger CV in the fractional case can be simply obtained by increasing the conductivity parameter *D*.

## Discussion

The results presented in this manuscript show that the introduction of a space-fractional operator in the monodomain formulation of the BR cell model affects both the temporal profile of the solution and the spatial propagation of the applied stimulus. Moreover, we identify well defined patterns in the value of a set of solution features corresponding to quantities of physiological interest as functions of the fractional order *α* of the differential operator in space. Specifically, we found a positive linear dependence from *α* of the depolarization peak height, a quadratic relationship between *α* and the CV, and a power law decay in the value of both the dome peak and the early repolarization minimum as *α* decreased. Furthermore, the analysis of the AP depolarization and the APD revealed a sensible increase in the AP foot width and a much more pronounced APD dispersion when the fractional order was reduced.

Although a direct comparison with experimental data is not provided in this work, the characteristics observed in the solution behaviour for *α* < 2, especially for AP foot shape and APD dispersion, are in agreement with the experimental data reported by Bueno-Orovio et al. in [11]. Standard excitable media models typically exhibit moderate dispersion of APD across the entire spatial domain (with an almost constant profile, except for the region in close proximity to a boundary). However, experimental studies on human, canine, and rabbit cardiac tissue (see [11] and references therein) exhibit a quite marked decreasing trend in the data relating the activation time of different locations of the tissue and the corresponding APD. Therefore, space-fractional models displaying a pronounced dispersion of APD as the repolarization front moves away from the stimulus site seem to be a better-suited modelling option to obtain consistent results with experimental data.

Similar conclusions can be made by examining the AP foot shape. Previous studies on the AP foot morphology [24, 25], have identified a direction-dependent behaviour of depolarization with respect to the orientation of the tissue fibres. These studies highlighted the presence of a prominent AP foot in the direction of longitudinal propagation (along the fibres) and linked the AP foot shape measured experimentally with the heterogeneous microscopic structure of cardiac tissue, showing that the AP foot width sensibly increases in the presence of a high capillary density in the tissue. This connection with the microscopic structure of the environment in which the pulse propagation occurs is in perfect agreement with the motivations behind the use of a space-fractional model for this application, and our results show that the value of *α* ∈ (1, 2] can be selected in order to account for a variety of AP foot widths (very narrow when *α* = 2 and increasingly larger as *α* decreases).

Another important aspect of our study concerned the identification of crucial differences between the solution of the standard formulation and of the space-fractional modification of the model. We highlighted the fact that the modification in the solution profile produced by the simple reduction of *α* allows us to account simultaneously for a wider AP foot and a more pronounced APD dispersion across the spatial domain, while the reduction in the conductivity parameter *D* simply affects the CV of the propagating excitation wave.

The study of the considered features of electrical wave propagation in two-dimensional and three-dimensional extensions of the space-fractional model proposed in this paper will be investigated in our future research.

## Conclusions

In this manuscript, we chose a simplified model of cardiac electrophysiology, namely the BR monodomain model, and considered its space-fractional modification on an insulated interval [0, *L*], by introducing in the model description the continuous one-dimensional fractional Laplacian (−Δ)^*α*/2^ of order *α* ∈ (1, 2] defined via its spectral expansion on the considered finite domain. In order to compute the solution to this space-fractional modification we developed a suitable variation of the numerical approach proposed by Whiteley [20] for the efficient solution of the monodomain equations. Specifically, we embedded in Whiteley’s strategy a spectral method to efficiently compute the solution of the space-fractional differential equation governing the transmembrane potential variable of the model. The proposed approach exploits the spectral definition of the differential operator (−Δ)^*α*/2^ on the insulated finite domain and incorporate in its definition the correct representation of insulating boundary conditions in both the standard and the purely fractional cases.

By computing the solution of the considered space-fractional modification of the BR monodomain model for multiple values of the fractional parameter in its domain of definition, we were able to establish specific patterns in the solution behaviour and in the value of a set of solution features of interest as functions of *α*. Furthermore, we provide good fitting functions for the quantification of these relationships.

The extension of this work to more elaborated excitable media models does not pose a challenge from the theoretical perspective and would only require additional effort in the implementation of a more complicated system of differential equations and the computation of its solution. The real limitation of the presented analysis is the fact that we only considered a one-dimensional finite domain. Experimental studies showing direction-dependent characteristics of the AP shape in two spatial dimensions, such as [25], seem to suggest the use of models with a different fractional order along each of the two spatial dimensions, namely the direction longitudinal to the cardiac fibres and the one transversal to the fibres, to account for the differences observed. However, addressing this point is not so straightforward. This is due to the lack of a firm theoretical basis for the restriction of space-fractional operators to finite domains with non-trivial boundary conditions in more than one spatial dimension. Overcoming this issue is not simple and although we find in the literature different definitions of space-fractional operators in multiple dimensions (for example, via the matrix transfer technique [26] or by using a sum of one-dimensional Riesz–Feller fractional derivatives defined as in [27]), a rigorous treatment of non-trivial boundary conditions in these cases has not been established yet. Nevertheless, we believe that the adoption of fractional models in electrophysiology is worth investigating in further detail and might offer valuable, innovative perspectives into the mechanisms governing electrical pulse propagation in highly heterogeneous media, such as the heart or the brain.

## Supporting Information

S1 Appendix The BR cell model. Complete mathematical description of the equations for the BR cell model as provided in the original work by Beeler and Reuter [14].
(PDF)

S2 Appendix Goodness of fit statistics. Here we report the analytic expression of the fitting curves introduced in the Results section, provide specific values for the parameters involved (with 95% confidence bounds), and use a set of statistics to evaluate how well the proposed curves approximate the data.
(PDF)
