# Supplementary material for: On the Order of the Fractional Laplacian in Determining the Spatio-Temporal Evolution of a Space-Fractional Model of Cardiac Electrophysiology
Source: PLoS One. 2015 Dec 2;10(12):e0143938. doi: 10.1371/journal.pone.0143938 (PMC4668072; doi:10.1371/journal.pone.0143938)
Supplement: S1 Appendix — Complete mathematical description of the equations for the BR cell model as provided in the original work by Beeler and Reuter [14]. (PDF) [file pone.0143938.s001.pdf]

S1 Appendix

The BR cell model

The BR model consists of a system of eight ordinary differential equations: one for the transmembrane potential v and seven for a vector of secondary variables $\mathbf{z} = (m, h, j, d, f, x, c)^T$. The six variables m, h, j, d, f and x are the gating variables of the model and $c = 10^7[\mathrm{Ca}]_i$ represent the intracellular calcium concentration and has been scaled to simplify the notation.

Due to the similar form of the six equations describing the evolution in time of the six gating variables, we formulate here the BR model only in terms of the following three equations:

$$C_m \frac{dv}{dt} + I_{\mathrm{ion}} = I_{\mathrm{stim}}, \tag{1}$$

$$\frac{dg}{dt} = \alpha_g(v)(1-g) - \beta_g(v)\, g, \tag{2}$$

$$\frac{dc}{dt} = 0.07(1-c) - I_s, \tag{3}$$

where equation (2) describes the evolution in time of the generic gating variable g $(= m, h, j, d, f, x)$ and the functions $\alpha_g(v)$ and $\beta_g(v)$ describe the channel opening and closing rates for the particular gating variable g to which the equation is referring to. For all six gating variables, both α_g and β_g have the form

$$\frac{C_1 \exp(C_2(v + C_3)) + C_4(v + C_5)}{\exp(C_6(v + C_3)) + C_7}, \tag{4}$$

where the values and the units of the constants involved are specified in Table 1.

The coupling term between equation (1) and the system describing the temporal evolution of the secondary variables in $\mathbf{z}$ is the ionic current $I_{\mathrm{ion}} = I_{\mathrm{ion}}(v, \mathbf{z})$. In the BR cell model the ionic current is the sum of four different components and according to the original formulation [1], it can be written as follows:

$$I_{\mathrm{ion}} = I_{\mathrm{Na}} + I_{\mathrm{K}} + I_x + I_s, \tag{5}$$

where I_{Na} is the current carried by sodium

$$I_{\mathrm{Na}} = (4m^3hj + 0.003)(v - 50),$$

I_{K} and I_x are potassium currents defined, respectively, by

$$I_{\mathrm{K}} = 0.35 \left\{ \frac{4(\exp(0.04(v + 85)) - 1)}{\exp(0.08(v + 53)) + \exp(0.04(v + 53))} + \frac{0.2(v + 23)}{1 - \exp(-0.04(v + 23))} \right\},$$

$$\text{and} \qquad I_x = 0.8\, x\, \frac{\exp(0.04(v + 77)) - 1}{\exp(0.04(v + 35))},$$

ms^{-1}	C_1 ms^{-1}	C_2 mV^{-1}	C_3 mV	C_4 $\text{mV}^{-1}\cdot\text{ms}^{-1}$	C_5 mV	C_6 mV^{-1}	C_7 –
α_m	0	0	47	-1	47	-0.1	-1
β_m	40	-0.056	72	0	0	0	0
α_h	0.126	-0.25	77	0	0	0	0
β_h	1.7	0	22.5	0	0	-0.082	1
α_j	0.055	-0.25	78	0	0	-0.2	1
β_j	0.3	0	32	0	0	-0.1	1
α_d	0.095	-0.01	-5	0	0	-0.72	1
β_d	0.07	-0.017	44	0	0	0.05	1
α_f	0.012	-0.008	28	0	0	0.15	1
β_f	0.0065	-0.02	30	0	0	-0.2	1
α_x	0.0005	0.083	50	0	0	0.057	1
β_x	0.0013	-0.06	20	0	0	-0.04	1

Table 1: Parameter values for the Beeler–Reuter model as given in [1].

and I_s is the calcium current given by

$$I_s = 0.09\, d\, f\, (v + 82.3 + 13.0287 \ln(10^{-7} c)).$$

In the equations above, all currents are in $\mu\text{A}\cdot\text{cm}^{-2}$, v is in mV, the six gating variables are dimensionless, $[\text{Ca}]_i$ is in moles per litre ($\text{mole}\cdot\text{l}^{-1}$), and time is expressed in ms.

References

[1] Beeler GW, Reuter H. Reconstruction of the action potential of ventricular myocardial fibres. J. Physiol. 1977;268:177-210.
