# Supplementary material for: On the Order of the Fractional Laplacian in Determining the Spatio-Temporal Evolution of a Space-Fractional Model of Cardiac Electrophysiology
Source: PLoS One. 2015 Dec 2;10(12):e0143938. doi: 10.1371/journal.pone.0143938 (PMC4668072; doi:10.1371/journal.pone.0143938)
Supplement: S2 Appendix — Here we report the analytic expression of the fitting curves introduced in the Results section, provide specific values for the parameters involved (with 95% confidence bounds), and use a set of statistics to evaluate how well the proposed curves approximate the data. (PDF) [file pone.0143938.s002.pdf]

S2 Appendix

Goodness of fit statistics

We report here the complete description of the fitting curves used in the main text to establish specific relationships between the fractional parameter α and four solution features of interest. The statistics used to evaluate the goodness of fit of each of the considered curves are:

1. the sum of squares due to error (SSE) measuring the total deviation of the response values from the fit to the response values

$$\text{SSE} = \sum_{i=1}^{n} (y_i - \hat{y}_i)^2 \,;$$

2. the square of the correlation between the response values and the predicted response values (R-square)

$$\text{R-square } = 1 - \frac{\text{SSE}}{\text{SST}}, \quad \text{where} \quad \text{SST } = \sum_{i=1}^{n} (y_i - \bar{y})^2 \,,$$

   with SST representing the sum of squares about the mean;

3. the adjusted R-square based on the residual degrees of freedom, that is,

$$\text{adjusted R-square } = 1 - \frac{\text{SSE}(n-1)}{\text{SST}(k)},$$

   where n is the number of response values and k is the number of residual degrees of freedom defined as $k = n - m$ with m equal to the number of fitted coefficients estimated from the response values;

4. the root mean squared error (RMSE) estimating the standard deviation of the random component in the data and defined as

$$\text{RMSE } = \sqrt{\frac{\text{SSE}}{k}}.$$

- Depolarization peak

  Linear fit:

$$f(\alpha) = p_1\,\alpha + p_2$$

  Coefficients (with 95% confidence bounds):

$$\begin{aligned} p_1 &= 8.917\ (8.819, 9.015) \\ p_2 &= -2.622\ (-2.776, -2.468) \end{aligned}$$

  Goodness of fit:

SSE: 0.01185	R-square: 0.9998
Adjusted R-square: 0.9998	RMSE: 0.03848

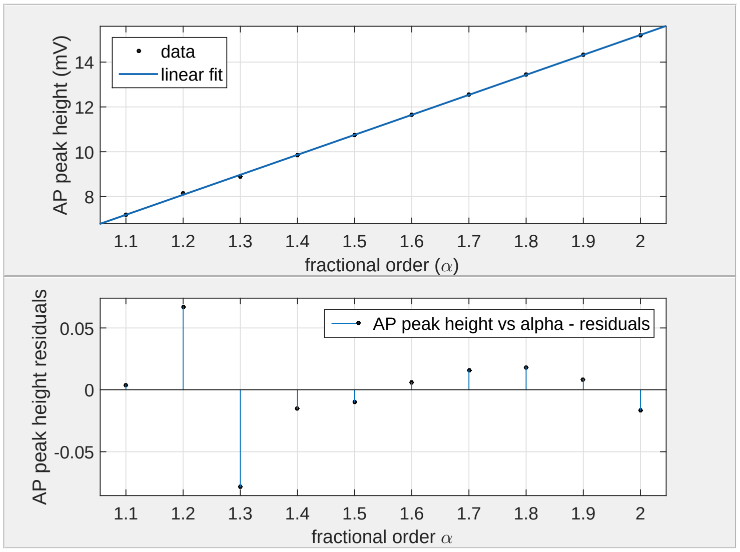

Figure 1: Curve fitting for the action potential peak height data and corresponding residuals.

- Action potential dome peak

  Power law fit:
  $$f(\alpha) = a\,\alpha^b + c$$
  Coefficients (with 95% confidence bounds):
  $$\begin{aligned} a &= -18.35\ (-18.75, -17.95) \\ b &= -5.037\ (-5.261, -4.813) \\ c &= 16.96\ (16.8, 17.12) \end{aligned}$$
  Goodness of fit:

SSE: 0.03155	R-square: 0.9997
Adjusted R-square: 0.9996	RMSE: 0.06713

- Early repolarization minimum

  Power law fit:
  $$f(\alpha) = a\,\alpha^b + c$$
  Coefficients (with 95% confidence bounds):
  $$\begin{aligned} a &= -47.51\ (-49.27, -45.75) \\ b &= -1.402\ (-1.504, -1.3) \\ c &= 24.37\ (22.34, 26.4) \end{aligned}$$

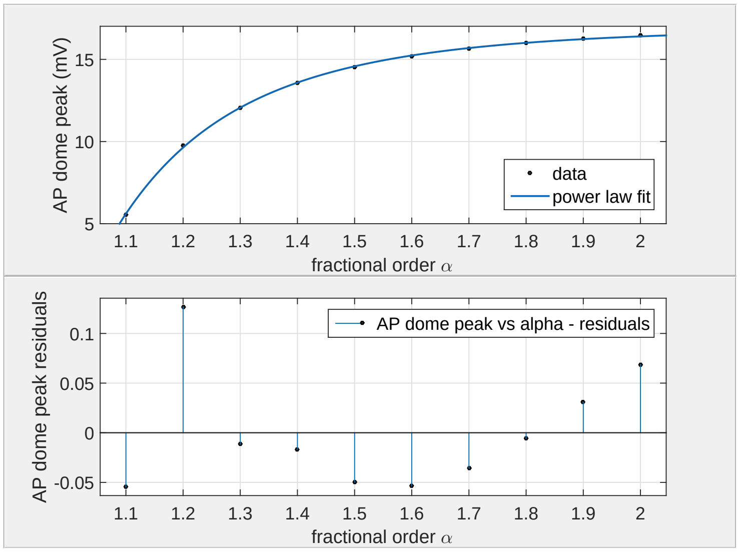

Figure 2: Curve fitting for the action potential dome peak data and corresponding residuals.

Goodness of fit:

SSE: 0.04996	R-square: 0.9999
Adjusted R-square: 0.9999	RMSE: 0.08448

- Conduction Velocity

  Quadratic fit:

$$f(\alpha) = p_1\alpha^2 + p_2\alpha + p_3$$

  Coefficients (with 95% confidence bounds):

$$\begin{aligned} p_1 &= 34.56\ (34.06, 35.07) \\ p_2 &= -65.18\ (-66.76, -63.61) \\ p_3 &= 32.34\ (31.14, 33.53) \end{aligned}$$

  Goodness of fit:

SSE: 0.01694	R-square: 1
Adjusted R-square: 1	RMSE: 0.04919

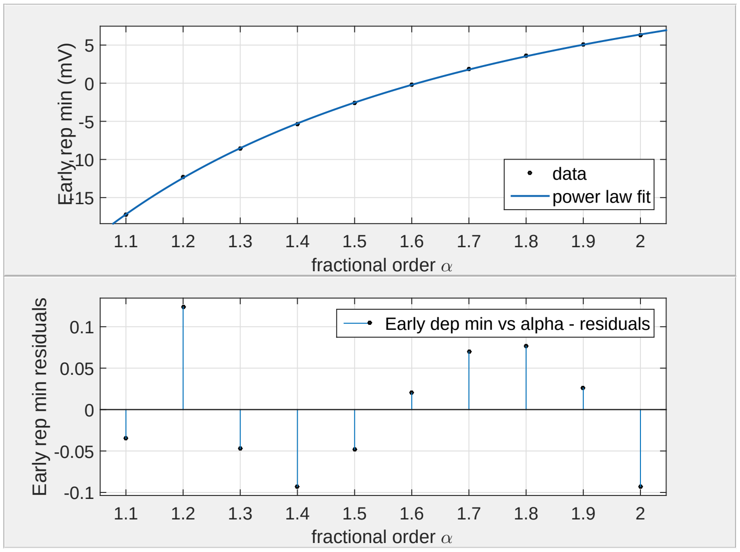

Figure 3: Curve fitting for the early repolarization minimum data and corresponding residuals.

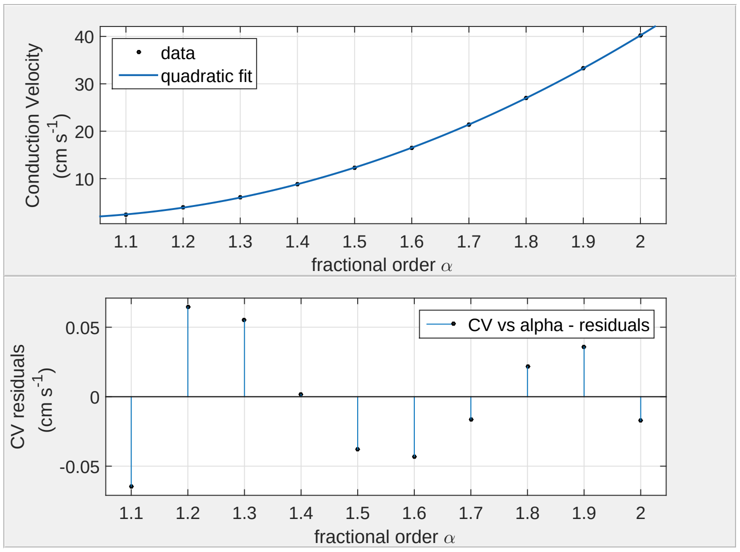

Figure 4: Curve fitting for the conduction velocity data and corresponding residuals.
